# Does Education Moderate the Relationship between Social Capital and Cognitive Function among Older Adults? Evidence from Suzhou City, China

**DOI:** 10.3390/ijerph17186560

**Published:** 2020-09-09

**Authors:** Jingyue Zhang, Nan Lu, Wenxiu Wang

**Affiliations:** 1 Institute of Gender and Culture, Changchun Normal University, Changchun 130032, China; zhangjingyue@ccsfu.edu.cn; 2 Department of Sociology, School of Philosophy and Sociology, Jilin University, Changchun 130012, China; 3 Department of Social Work and Social Policy, School of Sociology and Population Studies, Renmin University of China, Beijing 100872, China; 4 Sau Po Centre on Ageing, The University of Hong Kong, Hong Kong, China; 5 Department of Population, Resources and Environment, Northeast Asian Studies College, Jilin University, Changchun 130012, China; zjyz13@mails.jlu.edu.cn

**Keywords:** Social capital, cognitive function, older adults, urban China

## Abstract

While social capital is recognized as an important protective determinant of cognitive function in later life, there is a lack of research examining the potential moderators and mediators in the mechanisms linking social capital to cognitive function. This study investigated the moderating role of education on the relationship between social capital and cognitive function among older adults in urban Chinese communities. Data were derived from a community survey conducted in Suzhou, Jiangsu Province, China, in late 2015. A quota sampling method was applied to recruit respondents aged 60 years or older from 16 communities in the Gusu district. The final analytic sample size was 446. Multiple group analysis was applied to test the proposed model. The results show that cognitive social capital was significantly associated with cognitive function in the high education group only. Structural social capital was not significantly associated with cognitive function. The findings highlight the important role of social capital in influencing cognitive function in later life. Social capital interventions could be particularly useful as a preventive approach to help older adults sustain their cognitive function levels. Policy and intervention implications are discussed.

## 1. Introduction

The number of Chinese adults aged 65 years or older reached 166.58 million in 2018, accounting for 11.9% of the total population [1]. The urbanization rate increased from 49.95% in 2010 to 59.58% in 2018, indicating that a large proportion of the older Chinese population will live in urban communities in the next few decades [1]. At the same time, the number of oldest-old Chinese adults, aged 80 years or older, is growing rapidly and is estimated to constitute around one fourth of the oldest-old population worldwide in 2050 [2]. It is important to note that the prevalence of cognitive impairment and dementia increases with age. Under such circumstances, these conditions can lead to huge social and economic burden for individuals, families, and Chinese society. Identifying social determinants of cognitive function, which is defined as multiple mental capacities (e.g., memory and mental intactness such as numerical ability and time orientation) [3], is crucial to gain an in-depth understanding of why certain people are particularly vulnerable to cognitive impairment and dementia and to develop preventive interventions and strategies to slow down cognitive decline processes.

The concept of cognitive reserve is related to the levels of brain function and plays an important role in delaying the onset and reducing the rate of progression of cognitive impairment [4,5]. This concept highlights individuals’ abilities to handle stress and challenges to delay age-related cognitive decline. For those with healthy cognitive function, cognitive reserve plays an important role in promoting cognitive performance to deal with difficult tasks [5]. Insufficient cognitive reserve, however, might lead to greater difficulties in coping with stressors resulting from impaired memory or executive function [5,6]. Education and employment are two important social determinants of cognitive reserve [7,8]. Social capital is considered another important domain of reserve [4]. Supportive social network structure and social participation were found to be significantly associated with higher levels of cognitive function and lower cognitive decline rates in old age. However, there is a lack of research systematically investigating the interplay among these social determinants and their influences on cognitive function in later life. This study aimed to fill this research gap and investigated the moderator role of education on the relationship between social capital and cognitive function among older adults in an urban Chinese context.

### 1.1. Social Capital and Cognitive Function

Social capital can be defined from either a collective or individual perspective. According to Putnam’s conceptualization, social capital refers to “features of social organization, such as trust, norms, and networks, that can improve the efficiency of society by facilitating coordinated actions” [9]. Furthermore, social capital is defined as a formal of capital and an important social resource from individuals’ social connections, which feature common memberships, cultural values, and social norms [10,11]. Community is an important source of social capital in later life. Given that individual-level variation in social capital indicators (e.g., social participation and informal reciprocity) influence access to information sharing and the utilization of service and amenities, we assessed community-based social capital (hereafter social capital) at the individual level in this research. Moreover, social capital is a multidimensional concept, including cognitive and structural components. Cognitive social capital is defined as subjective evaluation of trust and reciprocity in neighborhood [12], whereas structural social capital refers to objective aspects of social involvement in local communities, including but not limited to number of organization memberships, citizenship activities related to the collective interest, volunteering, and social participation [12,13].

Stressful events could have adverse consequences for cognitive function in older age through structural changes in the hippocampus [14]. Social capital provides older adults with important opportunities for intellectual stimulation through more social connections and complex social interactions in diverse settings. Sometimes, these tasks can be cognitively challenging, but they might help the brain to preserve cognitive skills and even improve cognitive performance [5,15]. Furthermore, the stress-buffering hypothesis proposes that social capital might mitigate these adverse consequences by promoting individuals’ coping strategies and supportive resources and reducing the use of less adaptive responses to stress such as smoking and alcohol use [14,16]. For example, social capital improves older adults’ access to eldercare services and amenities in the community [17], which could further reduce the likelihood of cognitive decline and dementia. Close social connections in local neighborhoods can facilitate information about home- and community-based services and sharing of older residents’ personal care needs. This is especially important for vulnerable groups to let their voices to be heard by local social organizations. This could allow local social organizations to further improve access to and qualities of amenities and services to meet individualized needs in local communities.

Previous studies have identified the important role of social capital in promoting health outcomes in later life in Chinese contexts (e.g., depressive symptoms, life satisfaction, and self-rated health) [12,16,18,19,20,21,22,23]. Literature has shown that low levels of social participation and social interaction and high levels of loneliness were associated with cognitive decline and dementia [15,24]. Murayama, et al. [25] suggested that communities with high social capital allowed older adults with cognitive decline and dementia to continue to live in local communities. Furthermore, Hikichi, et al. [26] suggested that individuals still benefit from active social interactions with those with whom they were not familiar. Two recent Chinese studies found that social contact, social participation, and neighborhood safety were significantly associated with cognitive function in China [27,28]. However, nonsignificant findings were also reported [26,27,29,30]. For example, the association between dense neighborhood tie (an indicator of structural social capital) and subjective dementia symptoms were not statistically significant among older men in Japan [30]. The association between social trust (an indicator of cognitive social capital) and cognitive function was not significant among adult population aged 50 years and older in China [27]. This might be partially because of the lack of examination of potential mediators and moderators (e.g., gender, education, and rural–urban disparity).

### 1.2. Moderation Role of Education

As previously discussed, education is an important determinant of cognitive reserve [4,5]. Education plays an important role in providing intellectual stimulation over the life course [7,8]. Moreover, older adults with low educational attainment tend to engage in more unhealthy behaviors (e.g., smoking and drinking) than their counterparts. Education was also found to be significantly associated with structural social capital among older adults in urban Chinese contexts [31]. Educated older residents might use social capital in a more efficient manner, particularly in terms of obtaining health knowledge and information and accessing health services, which could be useful for them to prevent cognitive decline and the onset of dementia (e.g., physical activity) [15]. Bourdieu [11] proposed a dependence model of social capital, economic capital, and cultural capital. Individuals with low socioeconomic status not only possess lower levels of social capital, but they also use social capital effectively to meet their personal and collective interests.

According to this literature review and theoretical framework [9,10,12], we hypothesized that education would play a moderating role in the relationship between social capital and cognitive function among older adults in urban Chinese contexts. In specific, the association between social capital and cognitive function would be statistically significant in older adults with relatively high levels of educational attainments.

## 2. Materials and Methods

### 2.1. Sampling

This secondary data analysis was based on a cross-sectional data from a community survey collected in the district of Gusu, Suzhou, Jiangsu Province in China in 2015. Quota sampling approach was adopted to recruit community-dwelling older adults age 60 and older. Gusu is the central district in Suzhou. The local population numbered around 1 million at the time of the survey, around one fifth of whom were aged 60 years or older. Many of the residents had lived in the community for more than one decade. Around 90% owned their homes. Ethical approval was obtained from the Ethics Committee of the University of Hong Kong (EA1604030, April 28, 2016).

In China, district is the largest political division in a city. A district generally consists of multiple streets, and a street consists of multiple communities. In other words, community is the smallest administrative boundary and political division in a city. The sampling strategies are as follows: among the 16 streets of Gusu district, 1 to 2 communities were chosen from each street based on the recommendations from a local community center and committee on aging. From each community, 25 older respondents were selected. The criteria were as follows: the respondents should age 60 years or older, have a local household registration status, and live in local communities for more than half of year in 2015. Age and gender ratio were controlled and consistent with those from local representative sample based on the 6th national consensus.

Face-to-face interviews were conducted in the local communities. To control information bias (individuals with cognitive decline have difficulty accurately recalling their life events), this study only included cognitively healthy older adults. We also excluded older respondents with missing values on the education variable, generating a final sample size of 446. Research data are available in Supplementary Materials File S1.

### 2.2. Measurements

#### 2.2.1. Outcome Variable

We used the Short Portable Mental Status Questionnaire (SPMSQ) to measure cognitive function [32]. The Chinese version of SPMSQ has 10 items, and the scale’s validity has been established among older Chinese adults [33]. SPMSQ assesses older respondents’ cognitive function in multiple domains, including memory, calculation, orientation and personal history. In specific, the respondents were asked to answer questions about their age, current date (month and week), home location and address, serial subtraction of 3 from 20, the date of the mid-autumn festival, the date of the founding of People’s Republic of China, the first premier of the State Council in China, and the president of China. The responses were assessed by binary variables (0 = incorrect, 1 = correct). The summed scores were calculated (range = 0–10). Higher scores indicated higher cognitive function levels. The cutoff point of the scale was 7 for older respondents with college education or higher and 6 for those who completed high school education or had lower educational attainments.

#### 2.2.2. Social Capital Variable

We used trust and reciprocity variables to build the latent variable of cognitive social capital [13,34,35]. The respondents were asked to provide their opinions about the following four statements regarding (a) trust in the community: “You trust the majority of local residents in the community”; (b) a sense of belonging: “You consider the community as a family. You also consider yourself as a member of this family”; (c) willingness to collaborate with others: “The residents living in this community care about both their interests and others’ benefits”; and (d) perceived helpfulness of others: “The residents in this community help each other when necessary.” Answers were measured on a 5-point Likert scale (1 = strongly disagree, 2 = disagree, 3 = neutral, 4 = agree, 5 = strongly agree).

Organization memberships, citizenship activities, volunteering, and social participation were used to build the latent variable of structural social capital. First, the respondents were asked whether they had memberships in the following organizations in the last year: religious groups, women’s groups, political parties (the Communist Party of China and the Democratic Parties), sports groups, charitable organizations, neighborhood committees, labor unions, credit groups, and community associations. The answers were assessed by binary variables (0 = no, 1 = yes). Scores were summed up, ranging from 0 to 10. The respondents were also asked whether they participated in any volunteering activities in the last month (0 = no, 1 = yes) and the frequency of their social participation in activities held by the above organizations in the last year (ranging from 1 = never to 6 = more than twice per week). Furthermore, the respondents were asked the following statement: “Have you collaborated with other local residents to handle common problems in the last year?” (0 = no; 1 = yes).

#### 2.2.3. Moderator

Education was treated as a moderator in this study. The respondents were asked about their educational attainment (0 = illiterate, 1 = complete primary school education, 2 = complete secondary school education, 3 = complete high school education, 4 = complete college or university education and above). To examine the moderating effects of education on the relationship between social capital and cognitive function, education was recoded as a binary variable to form two groups (relatively low education group—complete primary school or lower; relatively high education group—complete secondary school education or higher).

#### 2.2.4. Covariates

Age was measured in years. Moreover, gender, living arrangement, marital status, and religion were recoded as binary variables (0 = men, 1 = women; 0 = living with others, 1 = living alone; 0 = other marital status, 1 = married; 0 = no religion, 1 = religious). The respondents were asked to report their household income per month and the number of their sons and daughters. Furthermore, financial satisfaction was assessed by a single question: “did you have adequate financial resources to cover your living cost in the past 3 months?” (0 = very inadequate, 2 = fair, 4 = very adequate). Finally, activities of daily living (ADLs) were assessed by the 10-item Barthel Index [36]. The items include eating, dressing, walking, getting out of bed/chair, going up and down stairs, watching face and brushing teeth, bladder control, going to toilets, continence of bowels, and bathing. Responses were measured on a 3-point scale (0 = very difficult, unable to complete independently; 5 = some difficult, need assistance; 10 = no difficulty, can complete independently). Summed scores were used to represent the levels of ADLs (range = 0–100). Higher scores indicated lower dependence in ADLs. Instrumental activities of daily living (IADLs) were assessed by the 7-item Lawton Instrumental Activities of Daily Living [37]. The items include preparing food, housekeeping, using transportation, using telephone, handling medication, handling finance, and shopping. Responses were measured on a 3-point scale (0 = very difficult; 1 = some difficult; 2 = no difficulty). Summed scores were used to represent the difficulty levels in IADLs. Higher scores indicated higher difficulty levels in IADLs.

### 2.3. Statistical Analysis

From a structural equation modeling (SEM) perspective, we used multiple-group analysis to test the moderation role of education in the association between cognitive social capital, structural social capital, and cognitive function [38,39]. SEM not only enables researchers to build latent variables with different coefficients in the associations between latent variables and observed indicators but also accounts for measurement error [39]. Multiple group analysis can be used to analyze models in different groups simultaneously. The two-step analytic procedures were as follows. First, the measurement models of social capital were tested by using confirmatory factor analysis in both education groups. We used a set of fit indexes to assess model fit, including chi-square test, Tucker–Lewis index (TLI), comparative fit index (CFI), root mean square error of approximation (RMSEA), and weighted root mean square residual (WRMR). The following criteria were used to determine a good model fit: non-significant chi-square estimate, TLI and CFI >0.95, RMSEA <0.05, WRMR < 1 [39,40]. Second, the factor loadings of social capital constructs were held equal across the two education groups. This approach allowed us to compare the regression coefficients across the two education groups. Third, we added cognitive function and covariates in the final model. We regressed cognitive function on the two latent variables of social capital, while controlling covariates. A Wald test was conducted to examine whether the moderating effects of education were statistically significant. Sensitivity analysis was conducted by applying different cutoff points and dividing older respondents into different education groups. The results were similar with the previous version. Therefore, we present the original findings. Mplus 7.0 (Muthén & Muthén, Los Angeles, CA, USA) was used to assist with the statistical analysis.

## 3. Results

### 3.1. Descriptive Statistics

We present the sample characteristics in Table 1. The respondents’ average age was 70.7 years. Of all respondents, 54.9% were women, 74.9% were married, and 17.5% lived alone. Around two thirds had completed secondary school education or higher. More than half of the respondents’ monthly household income was less than 5000 RMB ($726.78 USD). Furthermore, 90.6% and 87.7% of the respondents had no difficulties in performing ADL and IADL tasks, respectively; 79.8% answered all the questions correctly on the cognitive function test.

### 3.2. Multiple Group Analysis

The measurement model of social capital was tested in both low and high education groups. In the low education group, the estimates of fit indexes suggested good model fit (χ^2^(19) = 25.780, *p* = 0.1364, RMSEA = 0.048, CFI = 0.965, TLI = 0.948, WRMR = 0.571). The standardized estimates of factor loadings ranged from 0.530 to 0.792. The estimates of fit indexes also suggested good model fit in the high education group (χ^2^(19) = 29.943, *p* = 0.0525, RMSEA = 0.045, CFI = 0.970, TLI = 0.956, WRMR = 0.667). The standardized estimates of factor loadings ranged from 0.396 to 0.775. Factor loading invariance was established by holding factor loadings of social capital latent constructs equal across the two groups. The estimates of fit indexes also suggested that the model adequately fit the data (χ^2^(40) = 48.529, *p* = 0.1669, RMSEA = 0.031, CFI = 0.985, TLI = 0.978, WRMR = 0.902). We present the measurement model in Table 2.

**Figure 1:**
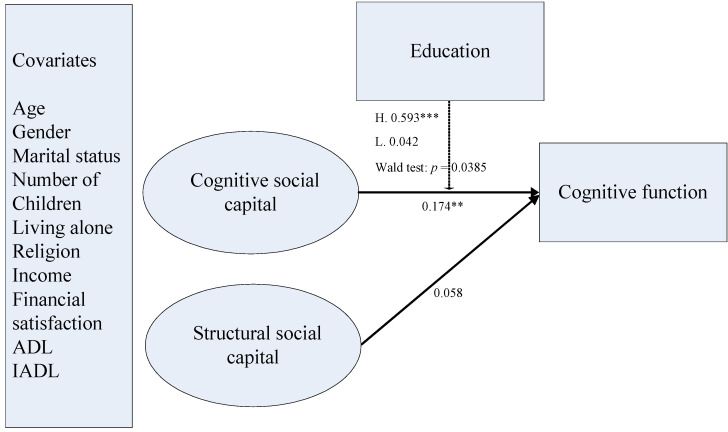
Final model of social capital and cognitive function; Notes: Standardized coefficients are reported. ** *p* < 0.01 (two-tailed); *** *p* < 0.001 (two-tailed); ADL = activities of daily living, IADL = instrumental activities of daily living.

In the next step, we added cognitive function and covariates in the final model. The estimates of fit indexes suggested a good model fit (χ^2^(176) = 194.470, *p* = 0.1618, RMSEA = 0.022, CFI = 0.969, TLI = 0.955, WRMR = 0.979). The associations between cognitive social capital and cognitive function were statistically nonsignificant in the low education group (unstandardized coefficient (*b*) = 0.088, *SD* = 0.239, *p* = 0.712). In contrast, in the high education group, cognitive social capital was significantly associated with cognitive function (unstandardized coefficient (*b*) = 0.632, *SD* = 0.110, *p* < 0.001). A Wald test was conducted to test the difference in the regression coefficients of cognitive social capital and cognitive function between the two education groups. The results identified a significant difference between the two groups (χ^2^(1) = 4.281, *p* = 0.039). The association between structural social capital and cognitive function was statistically nonsignificant in both education groups. We present the final model in Figure 1.

Additional analysis was conducted to test the relationship between social capital and cognitive function based on the whole sample (χ^2^(83) = 84.452, *p* = 0.4350, RMSEA = 0.006, CFI = 0.998, TLI = 0.997, WRMR = 0.611). Cognitive social capital was significantly associated with cognitive function (*b* = 0.338, *SD* = 0.114, *p* < 0.01). In contrast, the relationship structural social capital and cognitive function was nonsignificant (*b* = 0.050, *SD* = 0.061, *p* = 0.413).

## 4. Discussion

Sufficiently strong social capital, which can be characterized by high trust, strong reciprocity, dense social network, and frequent collaboration, could help older adults continue to independently live in local communities and handle life stressors efficiently, even when they encounter declines in their physical and cognitive health. The findings suggest that cognitive social capital is an important reserve domain in old age, especially for those with relatively high educational backgrounds. The findings added new empirical evidence for developing preventive interventions and strategies to sustain and promote cognitive health in urban China.

The majority of relevant studies used a single indicator to measure multi-dimensional social capital [12,26,27,28,29,30]. The present study used a SEM approach to conduct a more comprehensive and accurate instrument by establishing latent constructs of social capital among respondents with different education levels in urban Chinese settings. The selection of observed variables was consistent with the social capital questionnaires from World Bank, and recommendations from a recent systematic review of measurement of social capital in low- and middle-income countries [34,35]. The findings suggested that trust, reciprocity, organization memberships, and social involvement in community activities were considered important community assets and features for older adults to achieve healthy aging in local communities. High community social capital can provide older residents with a sense of security and belonging.

As discussed above, previous studies generated mixed findings in terms of the effects of cognitive social capital and structural social capital on cognition in later life [26,27,29,30]. This study added a new contribution by testing the moderation role of education in the above association. The findings of this study show that having higher levels of cognitive social capital was related to better cognitive function in the high education group. Structural social capital was not a significant determinant of cognitive function in both education groups. We conducted additional analysis based on the whole sample and found that structural social capital was significantly associated with cognitive function when cognitive social capital was not entered in the model. Furthermore, cognitive social capital and structural social capital were positively associated with each other. This indicates a potential mediator model in which structural social capital might affect cognitive function through cognitive social capital.

Social capital could influence cognitive function through multiple mechanisms. The stimulation from social interaction in a social environment and a healthy lifestyle, for example, could change brain structure and lead to improvements in the utilization of the brain network [5,14]. Furthermore, lack of social capital might lead to insufficient supportive resources in the community. Older adults consequently could have greater difficulty handling daily stress and challenges. In this case, a dense community network and strong social capital are more likely to contribute to the promotion of the quality of community-based services and amenities. Strong social capital was also associated with improved access to services and amenities [41]. These factors not only provide crucial supportive resources but also facilitate social interactions and reciprocity among older residents. These could benefit cognitive outcomes and delay the onset of dementia among local older residents. We argue that older adults with better educational backgrounds could utilize social capital more efficiently, which brings significant benefits to their cognitive function status.

The findings’ policy and intervention implications are as follows. First, community-dwelling older adults with relatively low education levels are at higher risk of poor cognitive function [7,8]. Therefore, this specific subpopulation deserves particular attention in future community-based social capital intervention development. Moreover, educational programs should be conducted to enhance the diffusion of information about health, nutrition, and self-management of chronic diseases among older adults. Volunteering programs should also be developed to encourage well-educated older adults to share their health-related knowledge and information with other local older residents. Third, older adults with different socioeconomic status might take different roles in both informal reciprocity in neighborhood and social participation in activities held by formal organizations. Future research and intervention should not only focus on solving potential barriers for older adults with low educational backgrounds but also design individualized and suitable interventions for older adults with different educational backgrounds.

The limitations of the present study are as follows. First, the study was based on the cross-sectional data. This means that we cannot examine the causal directions of the relationships among cognitive social capital, structural social capital, and cognitive function. The interplay between cognitive social capital and structural social capital was also not examined in this study. Second, random sampling was not used to collect data in this community survey. This limits the empirical generalization of the findings. Third, the key variables in this study were self-reported, which might cause information inaccuracy and misclassification bias. Finally, future qualitative inquiries are needed to gain in-depth understanding of the dynamics of social capital, cognitive function, and education in later life.

## 5. Conclusions

This study investigated the moderation effect of education on the relationship between social capital and cognitive function in later life in urban China. We established the latent constructs of social capital among both less-educated and well-educated older adults. The findings show that cognitive social capital was a significant determinant of cognitive function in the high education group only, whereas structural social capital was not significantly associated with cognitive function in both groups when cognitive social capital was entered in the final model. Social capital interventions might be particularly useful as a preventive strategy for older adults with relatively high educational backgrounds. Future social capital interventions should put great emphasis on the individualized needs among older adults with low educational attainment and help them utilize social capital in an efficient manner.

## Figures and Tables

**Table 1:** Sample characteristics (N = 446).

	N (%)	Mean (*SD*)
**Age**		70.7 (7.3)
60–74	319 (71.5)	
75 or above	127 (28.5)	
**Gender**		
Men	201 (45.1)	
Women	245 (54.9)	
**Married**	334 (74.9)	
**Be Religious**	88 (19.7)	
**Education**		
Primary school or lower	158 (35.4)	
Secondary school or above	288 (64.6)	
**Monthly Household Income**		
0–5000 RMB	241 → (54.1)	
>5000 RMB	201 (45.0)	
**Financial Satisfaction**		
Very inadequate/inadequate	38 (8.5)	
Very adequate/adequate/fair	408 (91.5)	
**ADL**		98.87 (4.7)
**IADL**		0.36 (1.5)
**Number of Children**		1.9 (1.1)
**Living Alone**	78 (17.5)	

Notes: 100 RMB = 14.54 USD; n = number; SD = standard deviation.

**Table 2:** Measurement model of social capital.

	Estimate	SD	Standardized Estimate	SD
**Low Education Group**				
Cognitive social capital				
Trust in local community	1.000	0.000	0.582 ***	0.054
Perceived helpfulness of others	1.549 ***	0.200	0.788 ***	0.053
Willingness to cooperate with others	1.106 ***	0.190	0.613 ***	0.077
Feelings of belonging	1.129 ***	0.145	0.666 ***	0.053
Structural social capital				
Organization memberships	1.000	0.000	0.540 ***	0.078
Volunteering	1.249 ***	0.253	0.814 ***	0.095
Social participation	1.349 ***	0.221	0.479 ***	0.072
Citizenship activities	1.055 ***	0.198	0.688 ***	0.095
**High Education Group**				
Cognitive social capital				
Trust in local community	1.000	0.000	0.462 ***	0.047
Perceived helpfulness of others	1.549 ***	0.200	0.699 ***	0.055
Willingness to cooperate with others	1.106 ***	0.190	0.518 ***	0.064
Feelings of belonging	1.129 ***	0.145	0.702 ***	0.049
Structural social capital				
Organization memberships	1.000	0.000	0.659 ***	0.063
Volunteering	1.249 ***	0.253	0.522 ***	0.076
Social participation	1.349 ***	0.221	0.744 ***	0.069
Citizenship activities	1.055 ***	0.198	0.579 ***	0.071

Notes: SD = standard deviation; *** *p* < 0.001 (two-tailed). *p* value is used to assess statistical significance level.

## Supplementary Materials

The following are available online at https://www.mdpi.com/1660-4601/17/18/6560/s1. File S1: Research Data.
